# Vascular nitrosative stress in hypertension induced by fetal undernutrition in rats

**DOI:** 10.1007/s13105-023-00949-1

**Published:** 2023-02-23

**Authors:** Pilar Rodríguez-Rodríguez, Anuson Poasakate, Santiago Ruvira-Hernando, Perla Y. Gutierrez-Arzapalo, Rainer Böger, Juliane Hannemann, Nicole Lüneburg, Silvia M. Arribas

**Affiliations:** 1 Department of Physiology, Faculty of Medicine, Universidad Autónoma de Madrid, C/ Arzobispo Morcillo 2, 28029 Madrid, Spain; 2 Food, Oxidative Stress and Cardiovascular Health (FOSCH) multidisciplinary research group, Universidad Autónoma de Madrid, Madrid, Spain; 3 Department of Physiology, Faculty of Medicine, Khon Kaen University, Khon Kaen, Thailand; 4 PhD student at Pharmacology and Physiology PhD Program, Doctorate School, Universidad Autónoma de Madrid, Madrid, Spain; 5 Present address: Centro de Investigación y Docencia en Ciencias de la Salud (CIDOCS), Universidad Autónoma de Sinaloa, Sinaloa, Mexico; 6 Department of Clinical Pharmacology and Toxicology, University Medical Center Hamburg-Eppendorf, Hamburg, Germany

**Keywords:** Nitric oxide, Fetal programing, ADMA, Nitrosative stress, Nrf2

## Abstract

Fetal undernutrition predisposes to hypertension development. Since nitric oxide (NO) is a key factor in blood pressure control, we aimed to investigate the role of NO alterations in hypertension induced by fetal undernutrition in rats. Male and female offspring from dams exposed to undernutrition during the second half of gestation (MUN) were studied at 21 days (normotensive) and 6 months of age (hypertension developed only in males). In aorta, we analyzed total and phosphorylated endothelial NO synthase (eNOS, p-eNOS), 3-nitrotyrosine (3-NT), and Nrf2 (Western blot). In plasma we assessed l-arginine, asymmetric and symmetric dimethylarginine (ADMA, SDMA; LC–MS/MS), nitrates (NOx, Griess reaction), carbonyl groups, and lipid peroxidation (spectrophotometry). In iliac arteries, we studied superoxide anion production (DHE staining, confocal microscopy) and vasodilatation to acetylcholine (isometric tension). Twenty-one-day-old MUN offspring did not show alterations in vascular e-NOS or 3NT expression, plasma l-Arg/ADMA ratio, or NOx. Compared to control group, 6-month-old MUN rats showed increased aortic expression of p-eNOS/eNOS and 3-NT, being Nrf2 expression lower, elevated plasma l-arginine/ADMA, NOx and carbonyl levels, increased iliac artery DHE staining and reduced acetylcholine-mediated relaxations. These alterations in MUN rats were sex-dependent, affecting males. However, females showed some signs of endothelial dysfunction. We conclude that increased NO production in the context of a pro-oxidative environment, leads to vascular nitrosative damage and dysfunction, which can participate in hypertension development in MUN males. Females show a better adaptation, but signs of endothelial dysfunction, which can explain hypertension in ageing.

## Introduction

Cardiometabolic diseases (CMD) continue being one of the leading causes of morbidity and mortality worldwide, despite efforts to modify inadequate lifestyle patterns (diet, sedentarism, smoking, and alcohol consumption), and the aid of pharmacological interventions targeting risk factors such as obesity, diabetes, or hypertension. In addition to the above-mentioned influences and the genetic background of the individual, the role of intrauterine life has emerged as a third factor contributing to the burden of CMD. It is now well established that exposure to various adverse environmental conditions during pregnancy and lactation (including nutritional disbalance, hypoxia, drugs, or toxics) are involved in the early-life programming of CMD [24]. In humans, the best characterized prenatal risk factor is fetal growth restriction (FGR) due to undernutrition or placental insufficiency, leading to low birth weight, a parameter associated with later development of CMD [13, 47]. The influence of adverse intrauterine environment has been confirmed with experimental animal models which mimic different stressors during gestation. These studies have been an invaluable tool to explore their effect on target organs, and the mechanisms implicated in this association.

Hypertension has emerged as one of the risk factors of CMD usually affected by exposure to adverse intrauterine environments, also observed in humans with FGR. Fetal adaptations to a suboptimal milieu result in alterations in key organs for cardiovascular control, such as the kidneys, the heart and the vasculature, changes which, in short term are adaptative, but in the long run become maladaptive [13, 47]. Studies in animal models exposed to various adverse factors evidence some common mechanisms and the sexual dimorphism in the response, being males more affected by suboptimal conditions during fetal and perinatal life [24, 14]. To explain this sexual dimorphism, the role of sex hormones in adult life [1, 16], as well as sex differences in placental development and its response to stressors have been proposed [10–36].

Nitric oxide (NO) is widely recognized as one of the key players in vascular homeostasis, balancing contraction-relaxation, proliferation-death, and the regulation of vascular function and structure. NO deficiency is a key pathogenic mechanism leading to CVD [40], and growing evidence implicates dysregulated NO system in the programming of hypertension and renal disease [24, 23]. Endothelial dysfunction is observed in various animal models exposed in utero to hypoxia [30, 43], nutritional disbalances [17–39], or toxic substances such as tobacco [3]. Therefore, endothelial dysfunction has been proposed as a relevant mechanism implicated in hypertension of developmental origin. However, there are some controversies on the role of NO in FGR pregnancies, with contradictory data in the literature [47]. NO local availability is controlled by the balance between generation and degradation. Synthesis is regulated by the accessibility of substrate, l-arginine (l-Arg), the expression and activity of NO synthases (NOS), and the levels of the natural NOS inhibitor asymmetric dimethylarginine (ADMA). ADMA and symmetric dimethylarginine (SDMA) are highly regulated endogenous molecules [22] that have been implicated in a variety of chronic CMD [7]. Elevated ADMA has been evidenced in pregnancies compromised by placental insufficiency and preeclampsia [2, 9], and ADMA exposure during gestation in rats induces hypertension in male offspring [24]. These data suggest that NO-ADMA disturbances may contribute to insufficient NO production and participate in fetal programming of hypertension. The other side of the equation contributing to NO availability as vasodilator is its degradation by reactive oxygen species (ROS), mainly superoxide anion, which is locally produced in the vascular wall by NADPH or other oxidases and by uncoupled NOS [15].

In an animal model of FGR induced by maternal nutrient restriction during the second half of gestation (maternal undernutrition, MUN), we have evidence that male offspring develops hypertension from adult age [19, 33], while females only exhibit moderate blood pressure elevation in ageing [20]. We hypothesize that vascular NO deficiency may be a possible underlying mechanism implicated in the development of hypertension in this model. To assess this hypothesis, we have analyzed different aspects of NO metabolism in male and female offspring at 21 days, a stage of life where the animals are still normotensive, and at 6 months, where only males have developed hypertension. Our specific aims were to analyze in vascular tissue and plasma relevant factors implicated in NO production (NOS protein expression, l-Arg and ADMA levels), and destruction (superoxide anion production, Nrf2 as antioxidant regulator, oxidative, and nitrosative damage), and NO-mediated vasorelaxation.

## Materials and methods

### Maternal undernutrition model

Experiments were performed in a model of fetal programming induced by maternal undernutrition (MUN) in Sprague-Dawley rats. The animals were obtained from the colony maintained at the Animal House Facility of the Universidad Autónoma de Madrid (ES-28079-0000097). All experimental procedures were approved by the Ethics Review Boards of Universidad Autónoma de Madrid and the Regional Environment Committee of Comunidad Autónoma de Madrid (RD 53/2013; Ref. PROEX 04/19).

The rats were housed in buckets (36.5/21.5/18.5 cm; length/width/height) on aspen wood bedding and maintained under controlled conditions (12/12 light/dark photoperiod, 22°C, 40% relative humidity), being health and welfare regularly monitored by the Animal House Facility staff. The rats were fed with a standard breeding diet (Euro Rodent Diet 22; 5LF5, Labdiet, Madrid, Spain) containing: 55% carbohydrates, 22% protein, 4.4% fat, 4.1% fiber, and 5.4% mineral.

The experimental model was established rats as previously described [33]. Twelve-week-old female rats were mated, and day 1 of gestation was determined by observation of sperm in the vaginal smear. Thereafter, the rats were allocated to one of the groups: Control (C, *n*=5 dams), or maternal undernutrition (MUN, *n*=5 dams), C dams received ad libitum diet throughout gestation and lactation. MUN rats received ad libitum diet during the first 10 days of gestation and 50% of the usual rat daily intake (previously estimated in 24 g/day) from day 11 to the end of gestation, returning to ad libitum diet during lactation. Drinking water was always provided ad libitum in both groups. Twenty-four hours after birth the pups were sexed and weighed individually, and the litter was standardized to 12 individuals, 6 males and 6 females if possible (smaller litters were not used). From each litter, some rats were studied at the age of 21 days (weaning) and others at the age of 6 months (adult), using 1–2 rats from each sex and age and the rest of the animals were aged for other studies. We have previously described that MUN male rats develop hypertension by the age of 6 months, assessed either by direct intra-arterial measurement under anesthesia or by tail-cuff plethysmography [33–41]. In the present study, tail-cuff plethysmography measurements from adult rats also showed that MUN males had significantly higher systolic blood pressure (SBP) compared to controls (control= 136.9±3.1 mm Hg, *n*=7; MUN=163.1±3.6, *n*=8; *p*=0.004). SBP in MUN females tended to be higher but did not reach statistical significance (control= 139.2±2.5 mm Hg, *n*=7; MUN=147.6±3.11, *n*=7; *p*=0.09).

### Experimental design

On the day of the experiment, the rats were first weighed. Thereafter, they were exposed to a CO_2_ chamber, and once rats were anesthetized, blood was obtained by cardiac puncture and killed by exsanguination. Blood was transferred to tubes with heparin (5000 units). Finally, the aorta and the iliac artery were immediately dissected. The aorta was snap frozen and stored at −70°C, and the iliac artery was kept at 4°C in Krebs-Henseleit solution (KHS) of the following composition: 115 mM NaCl, 4.6 mM KCl, 2.5 mM CaCl2, 25 mM NaHCO3, 1.2 mM KH2PO4, 1.2 mM MgSO4, 0.01 mM EDTA, 11 mM glucose, and used the same day. The blood was centrifuged for 10 min (900 g at 4°C) and the plasma was aliquoted and stored at −70°C until use.

### Plasma ADMA, SDMA, and l-Arg

The levels of ADMA, SDMA, and l-arginine were determined by liquid chromatography–tandem mass spectrometry (LC–MS/MS) as previously described [32]. Firstly, sample proteins were precipitated with methanol; a 96-well 0.20 μm-pore-size microfiltration plates pre-coated with l-[^2^H_7_] arginine, [^2^H_6_] ADMA, and [^2^H_6_] SDMA were used as internal standards. Thereafter, the samples were centrifuged, the microfiltrates were dried, and analytes were converted into their butyl ester derivatives. Finally, analyses were performed using a Chirobiotic T, 20 mm (long) × 1.0 mm (internal diameter), microbore guard column (Astec, Whippany, NJ; USA) connected to a Varian 1200L Triple Quadrupole MS instrument (Varian, Walnut Creak, CA; USA) in the ESI + (positive electrospray ionization) mode. The sample run time was 1.6 min and showed an intra-assay and inter-assay precisions of 2.2% and 4.7%, respectively.

### Plasma nitrates

Griess reaction, modified to microplate reader, was used. Briefly, a 100-μL plasma volume was incubated with 10 μL of N-ethylmaleimide 150 mM (Thermo Fisher Scientific, MA; USA) and 110 μL of trichloroacetic acid 20% w/v, and centrifuged (12,000 *g*, 5 min at 4 °C). A 40-μL volume of supernatant was transferred to a microplate and the following reagents were added: 40 μL of vanadium (III) chloride (saturated with hydrochloric acid 1 M), 20 μL of sulfanilamide 2% (w/v diluted in hydrochloric acid 5% v/v; Thermo Fisher Scientific, MA; USA), and 20 μL of naphthyl-ethylenediamine dihydrochloride 0.1% (w/v diluted in H2O-Q; Thermo Fisher Scientific, MA; USA). The mixture was incubated for 1 h at 37 °C, and the absorbance was read at 540 nm. Nitrates were expressed as μM.

### Plasma biomarkers of oxidative damage

The levels of carbonyls were evaluated in plasma as measurement of oxidative damage to proteins as previously described [31]. Plasma carbonyls were analyzed by a 2,4 dinitrophenylhydrazine assay, which detects protein carbonyls, adapted to microplate reader, using extinction coefficient of 2,4-dinitrophenylhydrazine (ε 22,000 M/cm). The absorbance was measured at 595 nm in a microplate reader, and the data were expressed as nmol/mg protein.

Oxidative damage to lipids was assessed by a LPO kit (Bioquochem; Gijón, Spain), which analyses MDA + HNE concentrations, as previously described [31]. The experiments were performed according to manufacturer’s instructions. The absorbance was measured in a microplate reader. MDA + HNE content was expressed as mM.

### Western blot

The aorta was used to assess the level of protein expression of eNOS, eNOS phosphorylated (p-eNOS, Ser 1177), 3-nitrotyrosine (3-NT), and Nrf2 by Western blot as previous described [31]. Firstly, the aorta was homogenized with lysis buffer of the following composition: 0.42 mM NaCl, 1 mM Na_4_P_2_O_7_, 1 mM DTT, 20 mM HEPES, 20 mM NaF, 1 mM Na_3_VO_4_, 1 mM EDTA, 1 mM EGTA, 20% glycerol, 2 mM phenylmethylsulfonyl fluoride (PMSF), 1 μL/mL leupeptin, 1 μL/mL aprotinin, and 0.5 μL/mL N-alpha-p-tosyl-l-lysine chloromethyl ketone hydrochloride (TLCK- hydrochloride). Thereafter, the tissue was centrifuged at 10,000 rpm, 4°C for 10 minutes, and the supernatant aliquoted and stored at −80 °C. Bradford assay (Bio-Rad, Pleasanton, CA, USA) was used to assess the level of proteins in the sample; absorbance was measured in a plate reader (Synergy HT Multi-Mode Microplate Reader, Biotek, VT, USA). To evaluate peNOS and total eNOS, 30 μg of protein per sample and a 7% SDS-PAGE gels were used. For 3-NT, 25 μg of protein and a 12% SDS-PAGE, and for Nrf2, 30 μg of protein and a 12% SDS-PAGE were used. Primary antibodies against p-eNOS (Cell Signaling Technology, USA; 1:250 final dilution), eNOS (BD Transduction, USA; 1:250 final dilution), 3-NT Abcam (Abcam, USA; 1:1000 final dilution), and Nrf2 (rabbit polyclonal, Abcam, USA; 1:1000 final dilution) were applied overnight at 4°C. After washing, secondary antibodies (anti-rabbit or anti-mouse IgG-peroxidase conjugated) were applied for 1 h. Blots were washed, incubated in commercial enhanced chemiluminescence reagents (ECL Prime, Amersham Bioscence, UK) and bands were detected by ChemiDoc XRS+Imaging System (Bio-Rad, USA). To ensure equal sample loading, all blots were re-incubated with monoclonal anti-β-actin-peroxidase (Sigma Aldrich; Spain, 1:10,000 final dilution). Blots were quantified using Image Lab 6.1 software (Bio-Rad, USA), and expression values of each protein were normalized with β-actin antibody.

### Assessment of endothelial function

Endothelial function was assessed in iliac arteries by isometric tension recording as previously described [35]. Briefly, 3-mm-long arterial segments were mounted on 168-μm-diameter iron wires and placed on organ bath chambers containing KHS, at 37 °C with carbogen gas (95% O_2_ and 5% CO_2_). The system was connected to a force transducer and a data registration system to monitor arterial tension (LabChart, AD Instruments, New Zealand). Arterial segments were adjusted to their optimal tension (1.5 g, obtained from previous experiments), stabilized for 30–40 min, readjusting tension and then exposed to 120 mM KCl to assess functionality (segments with responses below 0.5 g were discarded). To evaluate endothelium-dependent relaxation, the segments were pre-contracted with 10^−7^ M noradrenaline until stable contraction was obtained, and a concentration–response curve to acetylcholine (ACh, 10^−11^ to 10^−4^ M) was performed. The implication of NO in the response was assessed by ACh concentration-response curve in the presence of the NO-blocker NN-nitro-l-arginine methyl ester (l-NAME) 10^−4^ M after 20 min preincubation. All drugs and reagents were obtained from Sigma-Aldrich (USA). Stock solutions of drugs and reagents were dissolved in distilled water except dilutions of ACh and noradrenaline which were prepared in saline-ascorbic acid to avoid oxidation.

### Superoxide anion detection

The iliac artery was used to assess basal levels of superoxide anion generated in the arterial wall by dihydroethidium (DHE; Sigma-Aldrich; USA), as previously described [35]. Briefly, a 4-mm segment of the iliac artery was stabilized in KHS at 37 °C with continuous oxygenation using carbogen gas (95% O_2_ and 5% CO_2_) for 30 min. Thereafter, the artery was incubated with 3 × 10^−5^ M DHE prepared in KHS for 30 min under the same conditions in Eppendorf tubes wrapped in aluminum foil to ensure complete absence of light. The segment was then washed, fixed with 4% PFA for 1 h and stained with the nuclear dye DAPI (Life Technologies; USA) at 1:500 from stock 1 mg/mL, in the darkness at room temperature. The artery was washed 2 times (15 min each) and whole mounted on a slide provided with a well, made of silicon spacers, for confocal microscopy study. The arterial segments were visualized with Leica SP2 spectral confocal microscope under identical conditions of laser brightness, intensity, and contrast levels with a 40× objective with 2× zoom, using the 488 nm/590–620 nm line (DHE positive nuclei detection) and the 405/410–475 nm line (DAPI detection). Four different regions of the adventitial layer were scanned from each artery randomly chosen based on DAPI wavelength, and stacks of 15 serial optical slices (1-μm thick) were captured in each region and stored. Quantification was performed with FIJI free software counting the number of DAPI positive (all nuclei) and DHE positive nuclei (with the same threshold). The % of DHE positive nuclei from total nuclei was calculated and the average of the 4 regions analyzed in each segment was used for statistical analysis.

### Statistical analysis

Sample size was calculated assuming a probability error of alpha type of 5% (*p*<0.05) and potency of 80%. Statistical analyses were performed by Graph-Pad Prism (version 5) and SPSS (version 22). The data followed a normal distribution (analyzed with Kolmogorov–Smirnov test), and they were expressed as mean ± SEM. Statistical differences were assessed by 2-way ANOVA; considering group (MUN and control) and sex (males and females), also evaluating the interaction between sex and group. Significance level was established at *p*<0.05.

## Results

### Rat weight

MUN rats had significantly lower body weight at birth. This was observed both in males (C=6.48±0.14g; *n*= 24; MUN= 4.50±0.13g, *n*=24, *p*<0.001) and females (C=6.73±0.11g; *n*=24; MUN=4.72±0.13g; *n*=24; *p*<0.001). At the age of 21 days (weaning), body weight was not statistically different between MUN and control male (C=51.3±1.5g, *n*=24, MUN=50.6±1.27g, *n*=24) or female rats (C=51.3±1.5g, *n*=24; MUN=50.6±1.3g, *n*=24). At the age of 6 months, body weight was not statistically different between MUN and C males (C=504.5±15.1g, *n*=21, MUN=491.2±8.6g, *n*=22) or females. (C=301.4±6.8g, *n*=15, MUN=287.1±4.7g, n=18), being female weight significantly smaller compared to males in both experimental groups.

### Plasma l-Arg, ADMA, and SDMA

**Fig. 1:**
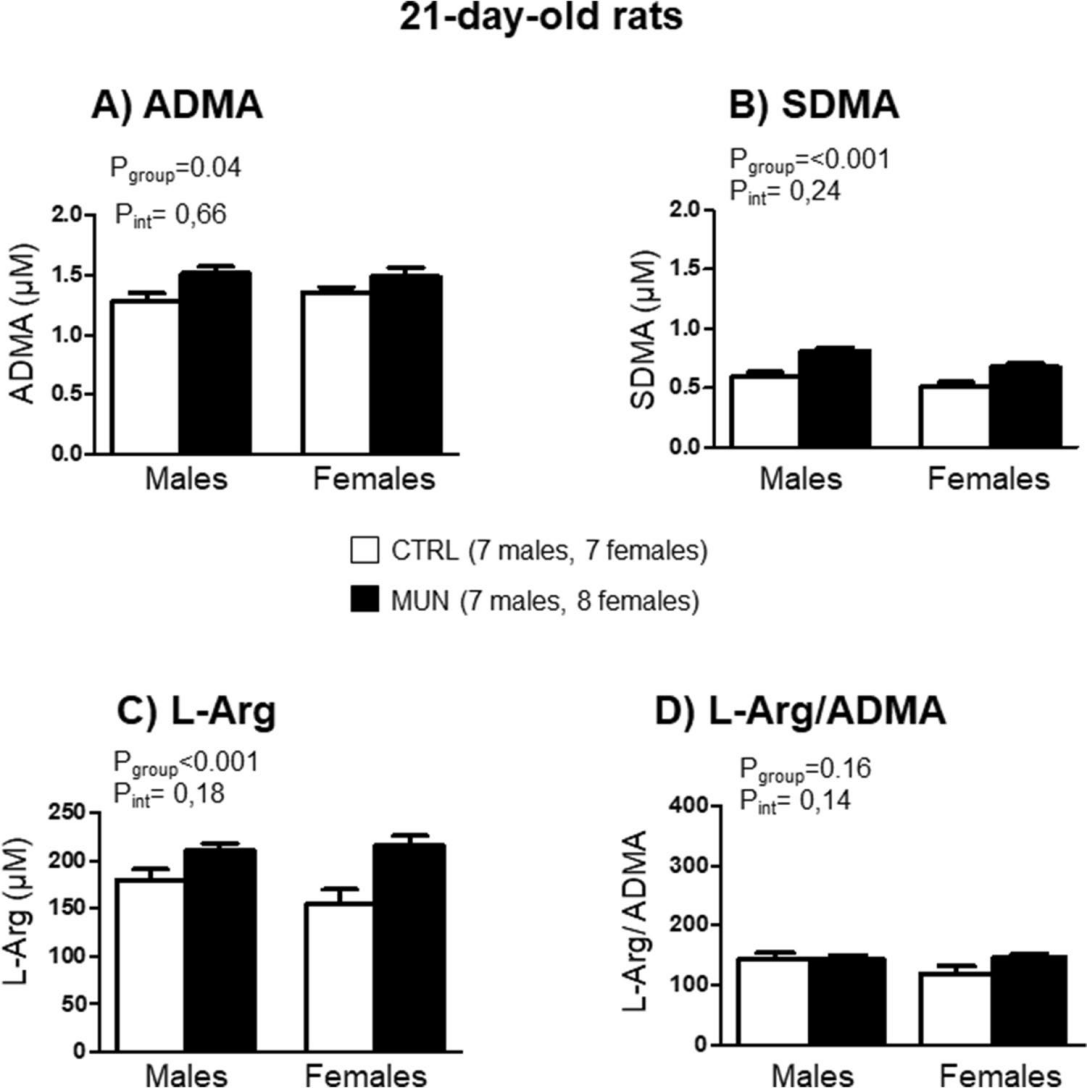
Plasma NO metabolites in 21-day-old rats. **A** ADMA, **B** SDMA, **C** l-arginine, and **D** l-Arg/ADMA in male and female offspring from rats exposed to maternal undernutrition during pregnancy (MUN) and rats fed ad libitum (control, CTRL). In parenthesis it is shown the number of rats from each experimental group and sex; each value is the average of duplicates. Data were analyzed by 2-way ANOVA; *p* values of group effect and the interaction between group and sex are shown in the figure. ADMA, asymmetric dimethylarginine; SDMA, symmetric dimethylarginine; l-Arg, l-arginine

At the age of 21 days, plasma ADMA, SMDA and l-arginine were higher in MUN group compared to control, with no statistical differences in the interaction between group and sex. l-Arg/ADMA ratio was not statistically different between MUN and control offspring. No significant difference in interaction was detected (Fig. 1).

**Fig. 2:**
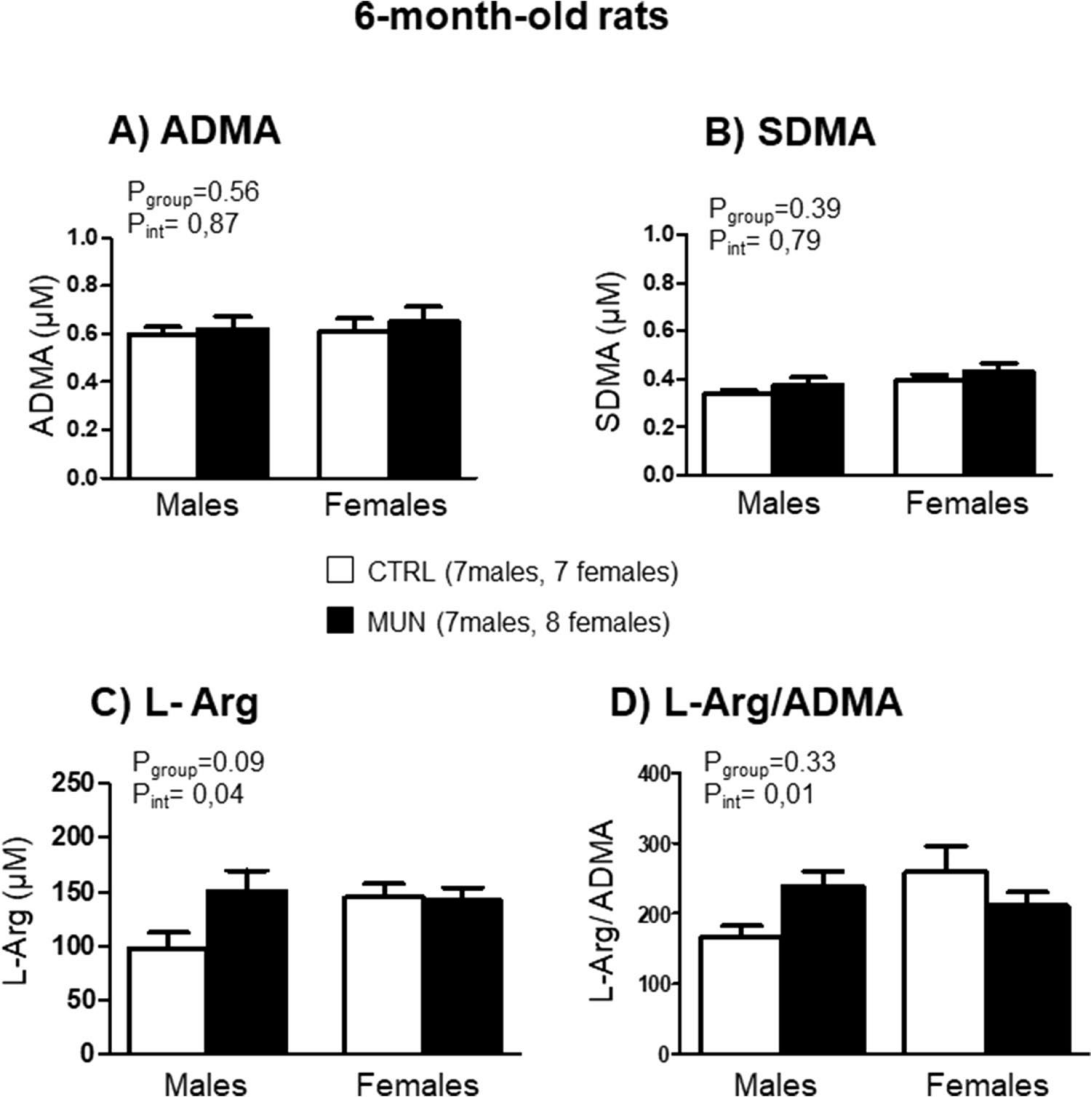
Plasma NO metabolites in 6-month-old rats. **A** ADMA, **B** SDMA, **C** l-arginine, and **D** l-Arg/ADMA in male and female offspring from rats exposed to maternal undernutrition during pregnancy (MUN) and rats fed ad libitum (control, CTRL). In parenthesis it is shown the number of rats from each experimental group and sex; each value is the average of duplicates. Data were analyzed by 2-way ANOVA; *p* values of group effect and the interaction between group and sex are shown in the figure. ADMA, asymmetric dimethylarginine; SDMA, symmetric dimethylarginine; l-Arg, l-arginine

At the age of 6 months, we did not find statistical differences in ADMA, SDMA between MUN and control or in the interaction between them. l-Arg and l-Arg/ADMA ratio did not show group differences. However, both parameters were higher in MUN males, and a significant interaction between fetal nutrition and sex was detected (Fig. 2).

### Plasma nitrate levels

**Fig. 3:**
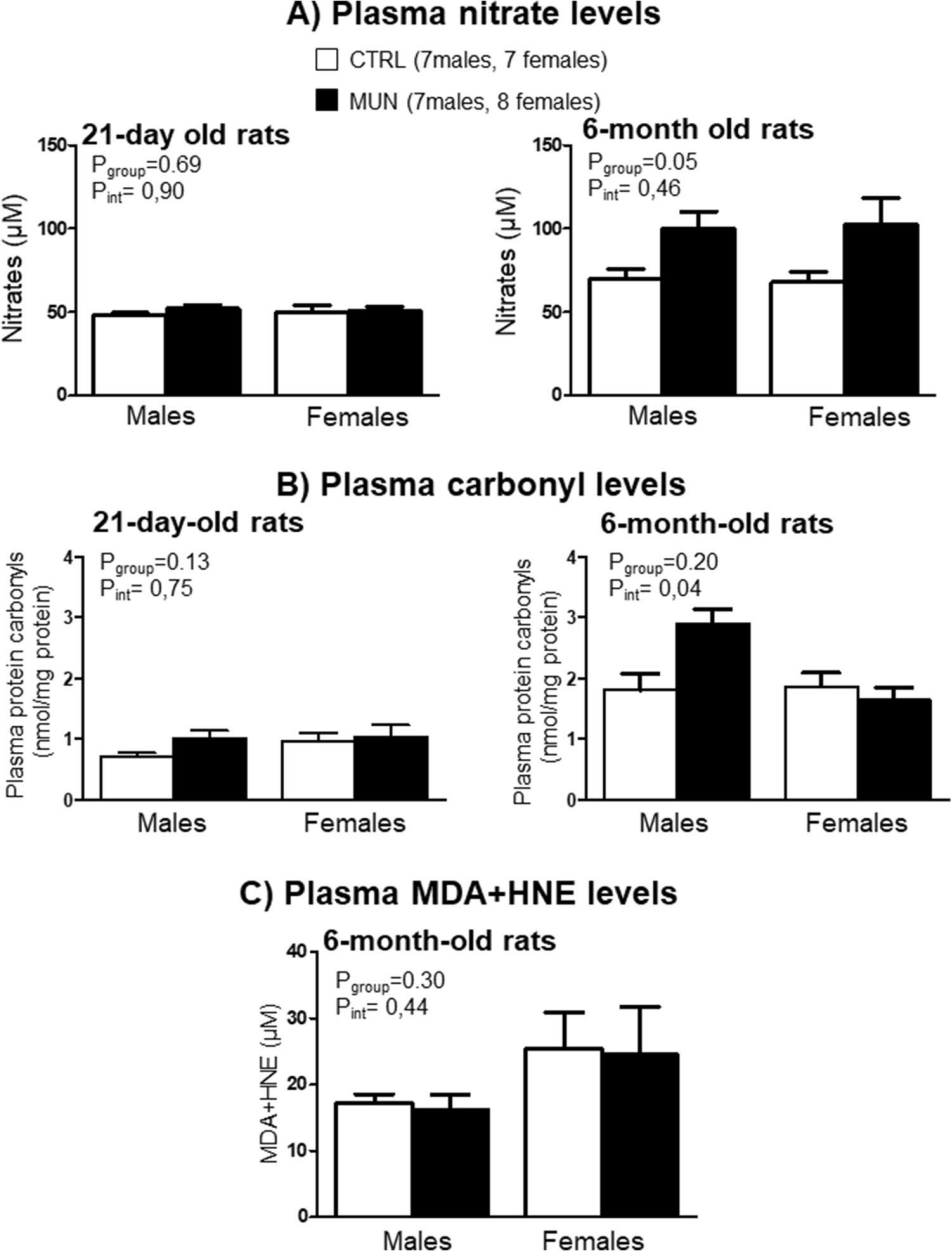
Plasma levels of nitrates (**A**), carbonyls (**B**), and MDA+HNE (**C**) in 21-day and 6-month-old rats. Male and female offspring from rats exposed to maternal undernutrition during pregnancy (MUN) and rats fed ad libitum (control, CTRL). In parenthesis it is shown the number of rats from each experimental group and sex; each value is the average of duplicates. Data were analyzed by 2-way ANOVA; *p* values of group effect and the interaction between group and sex are shown in the figure

Nitrates represent the final products of NO oxidation (nitrates and nitrites, NOx), and plasma NOx concentrations can be taken as an index of NO output [27]. At the age of 21 days, plasma nitrate levels were not statistically different between MUN and control groups and no significant interaction between group and sex was detected. At the age of 6 months, plasma nitrate levels were elevated in MUN groups, and no significant interaction between fetal nutrition and sex was detected (Fig. 3A).

### Plasma markers of oxidative damage

At the age of 21 days, plasma carbonyl levels did not show significant differences between groups or in the interaction between fetal nutrition and sex. At the age of 6 months, carbonyls were higher in MUN males and lower in MUN females compared to their controls, being significant the interaction between fetal nutrition and sex (Fig. 3B). At this age, we did not detect statistical differences in lipid peroxidation levels (MDA+HNE) between MUN and control rats or in the interaction between sex and group (Fig. 3C). It was not possible to perform experiments at the age of 21 days due to insufficient plasma sample.

### Protein expression in aorta

**Fig. 4:**
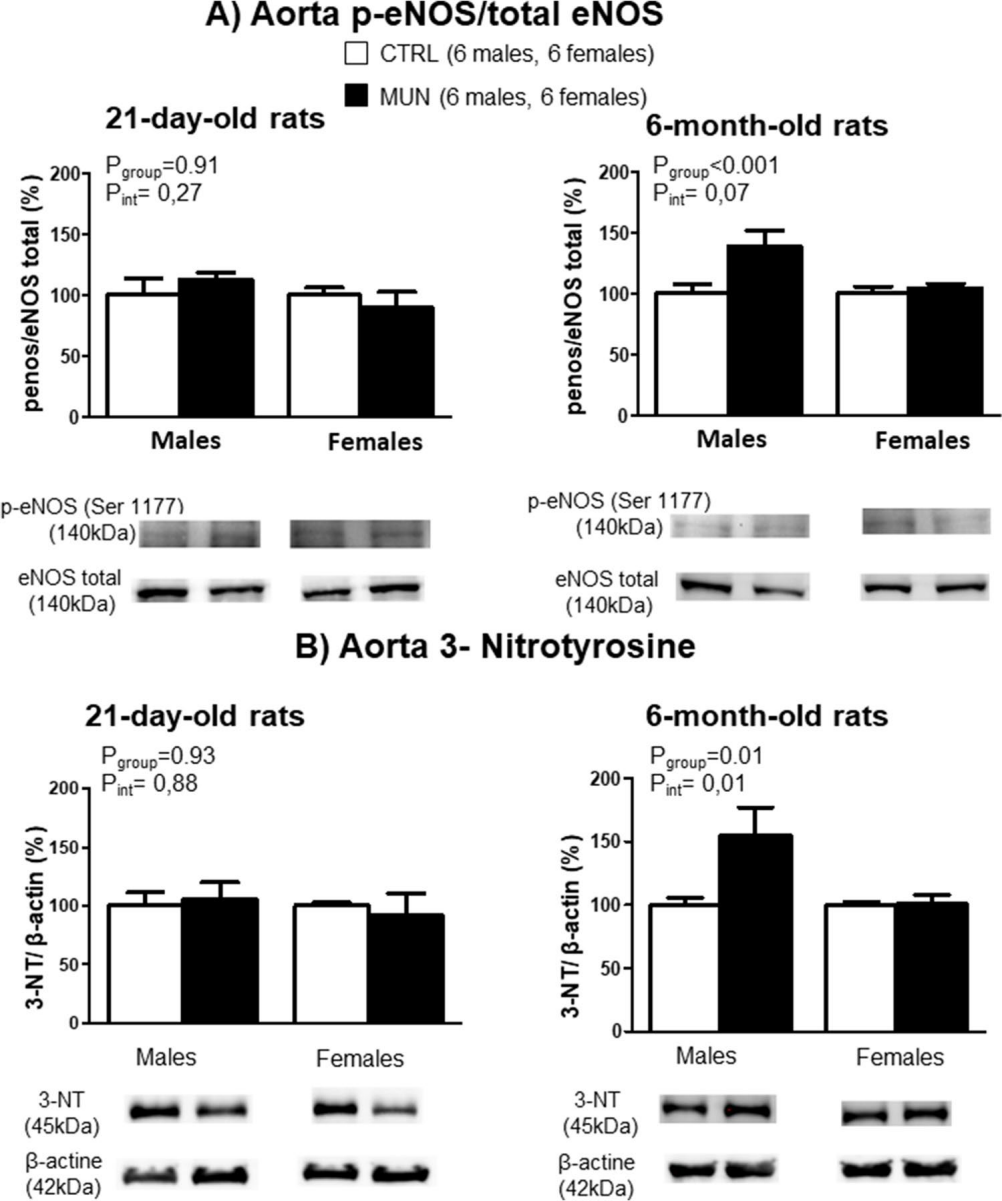
Aorta protein expression of p-eNOS/total eNOS (**A**) and 3-nitrotyrosine (**B**) in 21-day and 6-month-old rats. Male and female offspring from rats exposed to maternal undernutrition during pregnancy (MUN) and rats fed ad libitum (control, CTRL). Bar graphs show the results of densitometric analysis, relativized to total eNOS expression or β-actin, and representative western blot data are shown at the bottom of each graph. In parenthesis it is shown the number of rats from each experimental group and sex; data were analyzed by 2-way ANOVA; *p* values of group effect and the interaction between group and sex are shown in the figure; p-eNOS, phosphorylated eNOS; 3-NT, 3-nitrotyrosine

In the aorta from 21-day-old rats, there was no statistical difference in p-eNOS/eNOS expression between MUN and control group, or in the interaction between sex and group. At the age of 6 months, p-eNOS/eNOS was significantly higher in MUN groups, particularly in males. No significant interaction between fetal nutrition and sex was detected, but interaction was near statistical significance (*p* value=0.07) (Fig. 4A).

In the aorta from 21-day-old, rats no significant differences were observed in the levels of 3-NT between MUN and control rats. However, at the age of 6 months, the levels were significantly higher in MUN males, being the interaction between fetal nutrition and sex also statistically significant (Fig. 4B).

**Fig. 5:**
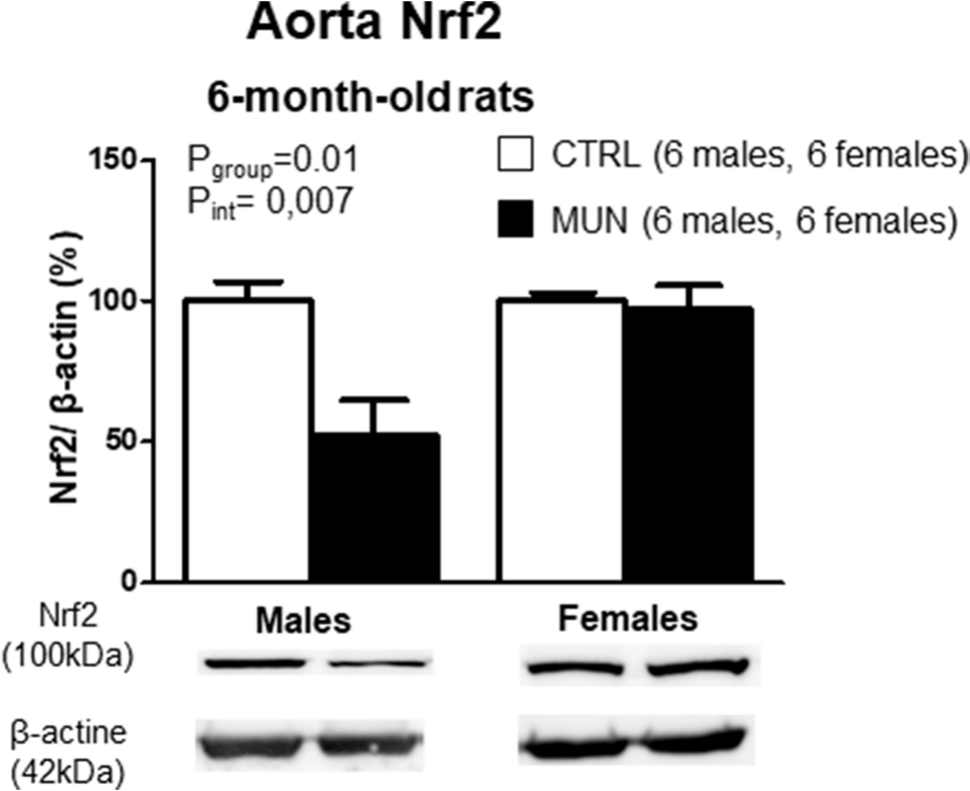
Aorta protein expression of Nrf2 in 6-month-old rats. Male and female offspring from rats exposed to maternal undernutrition during pregnancy (MUN) and rats fed ad libitum (control, CTRL). Bar graphs show the results of densitometric analysis, relativized to β-actin, and representative western blot data are shown at the bottom of the graphs. In parenthesis it is shown the number of rats from each experimental group and sex. Data were analyzed by 2-way ANOVA; *p* values of group effect and the interaction between group and sex are shown in the figure. Nrf2, nuclear factor erythroid 2-related factor 2

In adult rats, the levels of Nrf2 were significantly lower in MUN males, being significant the interaction between fetal nutrition and sex (Fig. 5).

### Study in iliac arteries

**Fig. 6:**
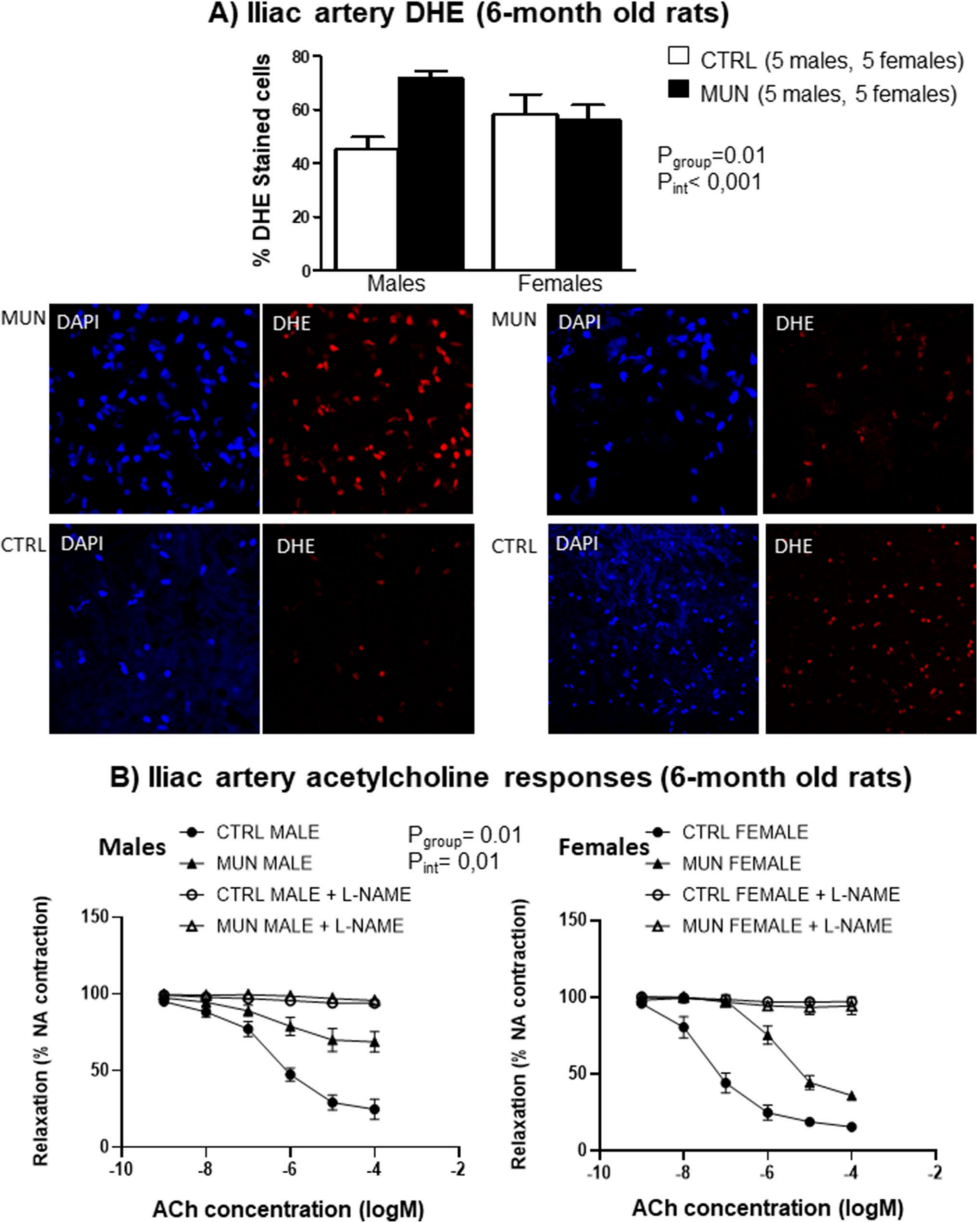
**A** Superoxide anion production and **B** acetylcholine concentration response curves in iliac arteries from 6-month-old rats. Male and female offspring from rats exposed to maternal undernutrition during pregnancy (MUN) and rats fed ad libitum (control, CTRL). **A** Bar graphs showing DHE-positive cells relative to total number of cells and representative confocal images of iliac arteries stained with DAPI and DHE (males, left panels; females, right panels). Images are reconstructions from a 10-μm-thick section of the adventitia taken with a 40× objective with 2× zoom. Image size 512 × 521 pixels. **B** Curves represent vascular relaxation to increasing concentrations of acetylcholine relative to previous contraction with 10^−7^ M noradrenaline. Data represent the average of 5 rats of each experimental group (2 segments from each rat). Data were analyzed by 2-way ANOVA, *p* values of group effect and the interaction between group and sex regarding maximal relaxation are shown in the figure. DHE, dihydroethidium; NA, noradrenaline; ACh, acetylcholine; l-NAME, NN-nitro-l-arginine methyl ester

In the iliac artery from adult rats (an artery with NO-mediated vasodilatation, similar to the aorta), we evaluated the basal release of superoxide anion and vascular responses. The number of cells stained by DHE (fluorescent marker of superoxide anion) were significantly higher in MUN males with respect to their sex-matched controls, while not differences were found between females, being significant the interaction between fetal nutrition and sex (Fig. 6A).

In iliac arteries, average contractions to KCl (unspecific vasoconstrictor) were not different between MUN and control groups. In males, responses were (control= 1.96 ± 0.70g, MUN= 1.57 ± 0.77 g; *p*=0.48), and in females, KCl responses were (control= 1.74 ± 0.26g, MUN= 1.62± 0.30 g; *p*=0.57). Noradrenaline 10^−7^ M precontraction was not significantly different between groups. In males, noradrenaline response was (control= 1.05± 0.072 g; MUN= 1.13 ± 0.18 g; *p*=0.11), and in females (control= 0.90 ± 0.07 g; MUN= 0.81 ± 0.13 g; *p*=0.16). Acetylcholine concentration response curve was significantly lower in MUN rats compared to controls (Fig. 6B). Maximal response in % relaxation to the noradrenaline precontraction was significantly lower in MUN groups. This response was lower in males (control= 72.41 ± 3.42%; MUN= 29.77 ± 0.77%) than in females (control= 81.16 ± 2.56%; MUN= 64.94 ± 2.82%), being significant the interaction between group and sex (Fig. 6B). In both sexes, ACh responses were completely abolished by l-NAME preincubation, which indicated they were NO-mediated (Fig. 6B). Basal contraction to l-NAME was significantly lower MUN males (control= 1.88 ± 0.21 g; MUN= 1.19 ± 0.12g), while no differences were detected in females (control= 1.30 ± 0.02 g; MUN= 1.34 ± 0.09g). Two-way ANOVA showed that basal l-NAME-induced contraction was significant for group (*p* value= 0.03) and interaction between sex and group (*p* value=0.02).

## Discussion

**Fig. 7:**
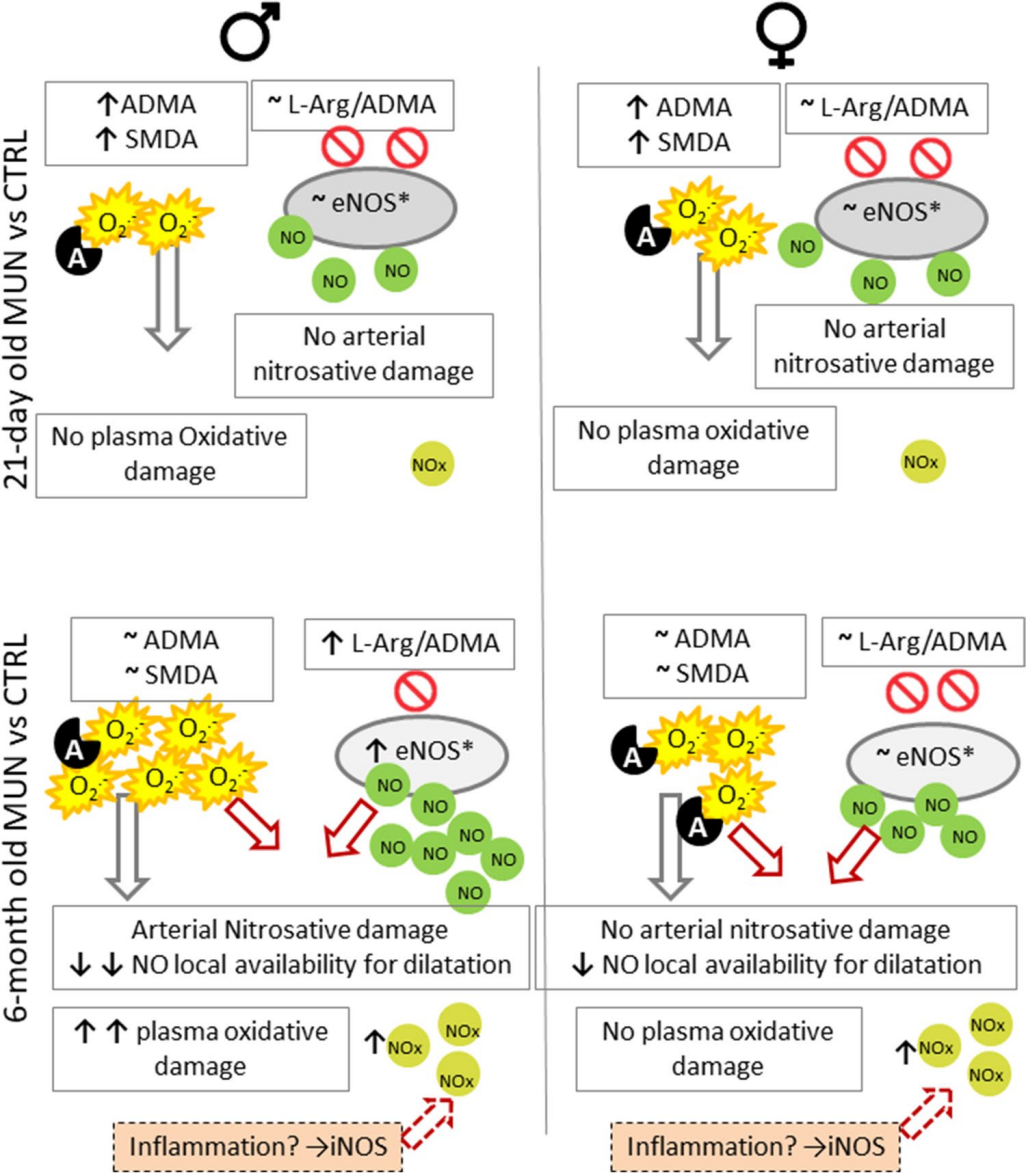
Schematic diagram depicting the main findings of the study. The figure shows the of alterations related to NO metabolism, oxidative and nitrosative damage from weaning period (21 days) to adult life (6 months) and the comparison between males and females. CTRL, control; NO, nitric oxide; eNOS*, active endothelial Nitric Oxide synthase; NOx, plasma nitrate levels; O_2_^.´^, superoxide anion; A represents antioxidants, likely low in 6-month-old MUN males, as suggested by the lower Nrf2. In the diagram, we also propose iNOS from extravascular cells activated by inflammation as potential source of elevated plasma NOx

In the present study, we aimed to evaluate the role of NO alterations in fetal programming of hypertension induced by maternal undernutrition during gestation, which we have previously demonstrated in adult male offspring [19–34], and was confirmed in the present study. Our main findings are that, at weaning, before hypertension development, MUN rats do not show alteration in vascular e-NOS expression, l-Arg/ADMA ratio or NOx in the circulation, suggesting no major alterations in NO release. Besides, plasma oxidative biomarkers or vascular nitrosative damage were not detected. In adult MUN males, elevation of active eNOS expression together with higher l-Arg/ADMA ratio was observed, suggesting higher NO release. However, this enhanced NO production was associated with nitrosative damage in the aorta, oxidative damage in plasma, and elevation of superoxide anion production in iliac artery. The above alterations were sex-dependent, suggesting that in MUN males, an elevated NO and ROS lead to vascular tissue nitrosative damage. Besides, a marked endothelial dysfunction was observed in MUN male iliac artery, and although MUN females also showed a lower vasorelaxation, endothelial dysfunction in MUN rats also had a sex-dependent component. Our data suggest that an elevation of NO production in the context of a pro-oxidative environment, leads to vascular injury and is a contributing factor to hypertension development in MUN males. We also detect some degree of endothelial dysfunction in MUN females, which could contribute to the higher blood pressure levels we have previously reported in old age [20]. A graphical summary of the main results is shown in Fig. 7.

NO deficiency has been proposed to play a role in hypertension of developmental origin evidenced by high blood pressure in offspring from eNOS KO mice [12]. Besides, inhibition of NOS by ADMA during gestation also results in blood pressure elevation in adult offspring [24]. Based on these facts, we aimed to evaluate the possible implication of NO balance in the development of hypertension in male rats exposed to nutrient restriction in fetal life, analyzing two age points, 21 days and 6 months. We studied the weaning period, since it may be a critical window where alterations initiated during intrauterine life, consolidate. This is supported by human data evidencing that endothelial dysfunction is already observed in childhood in individuals with low birth weight due to FGR, setting the background for the development of hypertension later in life [47]. MUN rats are born with low weight and during lactation they experience a catch-up growth, leading to similar body weight compared to controls. We also have evidence that catch-up growth process is deleterious leading early hypertrophy of adipose tissue [28], the heart [34], and aorta [19]. In humans with FGR, there is also evidence that postnatal accelerated growth has negative consequences for cardiometabolic health [13]. In addition, at weaning, both male and females are still normotensive [33, 34]. Therefore, exploring the age of 21 days enables to evaluate if early alterations in NO metabolism, prior to hypertension development, may contribute to blood pressure elevation in males in adult life.

NO bioavailability is complex and regulated by the balance between synthesis and degradation, in turn, both are controlled by various factors. In weaning rats, we did not detect alterations in the levels of active eNOS protein expression, assessed as the phosphorylated to global eNOS ratio, or in the degree of eNOS inhibition, evaluated as l-Arg/ADMA ratio [7, 6]. In accordance, in young animals, no alterations in plasma nitrates, the final products of NO oxidation (NOx), and representative of NO output [27] were detected. Although l-Arg/ADMA ratio was not different between groups, ADMA and SMDA were elevated in 21-day-old MUN offspring. ADMA and SDMA are released from tissues during physiological turnover of di-methylated proteins [21]. ADMA is enzymatically inactivated by dimethylarginine dimethylaminohydrolase (DDAH) [22] whilst SDMA is cleared by the kidneys. It was previously reported that dysfunction of the placental ADMA metabolism by DDAH contributes to high ADMA concentration in the late phase of pregnancy and may be one common cause of preeclampsia and FGR [2, 9]. Dysfunction of DDAH may be caused by genetic polymorphisms [22] or by oxidative inactivation of the enzyme [21]. It is possible that the higher ADMA levels observed at weaning could be related to oxidative inactivation of DDAH, as we have previously demonstrated a lower degree of antioxidants in MUN rats at this age, particularly in males [33]. Besides, it must be noted that elevated ADMA can further increase ROS levels uncoupling eNOS via depletion of BH4 and producing superoxide [38]. Therefore, we cannot rule out the role of an early rise in ADMA in the progression towards oxidative stress. Plasma ADMA elevation has also been reported in premature infants, who are deficient in antioxidants, and also have higher risk cardiometabolic diseases [38]. SDMA was also higher in 21-day-old MUN rats. SDMA, does not directly inhibit NOS [38], and, therefore, it is not likely that it would have an effect on NO levels. However, elevation may be deleterious, since, similar to ADMA [8], SMDA is an independent risk marker for all-cause mortality and cardiovascular disease [29], being both uremic toxins [38].

In adult rats, we found an upregulation of active e-NOS in the aorta from MUN males. Similar results have been found in models of maternal undernutrition [18] and fetal hypoxia [46], while other study reported a decreased eNOS RNA expression in the aorta from undernourished rats [17]. We propose that this difference may be related to the more severe nutrient restriction of Franco’s study including the entire pregnancy period. The influence of stress factors on different pregnancy stages have also been evidenced in human populations exposed to starvation, such as during the II World War. Offspring from mothers exposed to undernutrition during early gestation experienced elevated rates cardiometabolic diseases, those exposed in mid-gestation evidenced reduced renal function and no effect was observed in late gestation [24]. Taken these data together, it is relevant to consider gestation period to interpret the results in animal models and to consider this aspect in the context of human pregnancy and fetal programming.

NO is eliminated by superoxide anion produced in the vascular wall, decreasing its bioavailability for vasodilatation. We used DHE, a dye that combines with superoxide anion generating ethidium bromide, which binds to DNA, and stains nuclei. MUN rats had a larger DHE staining, being significant the interaction between group and sex. Our data suggest a higher vascular release of superoxide anion in MUN males. Although DHE is an indirect measurement and this technique has some limitations, to avoid bias we ensured the same experimental conditions for all the tissues, including incubation, image capturing, and quantification. This effect was sex-dependent and may be related to a higher superoxide anion production and/or to a lower elimination by antioxidant enzyme systems, since we have evidence that male rats of MUN model are deficient in antioxidants [33, 34]. The smaller antioxidant capacity of MUN males was also evidenced by the low Nrf2, a factor that regulates adaptive response to oxidative stress promoting the transcription of antioxidant enzymes [4], and is downregulated in hypertensive animals [26]. The excess superoxide anion may be due to increased NADPH oxidase in the arterial wall, as we have demonstrated in mesenteric arteries from MUN males [41]. Besides, it can be due to eNOS uncoupling, leading to superoxide anion, which has been observed in other models of hypertension programming [18, 46]. These studies also report an increased eNOS expression, as observed in the present study, but reduced local NO release. We could not assess local NO, but based on our data and previous observations, we propose that a local NO deficiency due to destruction may be a stimulus for upregulation of the eNOS enzyme. A similar compensatory mechanism has been previously described in pregnancies complicated with FGR, where a higher eNOS expression was found in the placenta, as compensatory mechanisms to improve placental blood flow [47].

The excess NO and superoxide anion locally produced in the vascular wall are deleterious, evidenced by the high level of 3-NT in the aorta in MUN males. As observed with DHE staining, 3-NT expression was higher in arteries from MUN males; the influence of sex was evidenced by the significant interaction between group and sex. NO is a free radical, which readily interacts with superoxide produced in the vicinity leading to peroxynitrite, a very reactive and unstable molecule, which quickly decomposes when protonated to form potent oxidants (hydroxyl radical and nitrogen dioxide), with high oxidative capacity [15]. The cytotoxicity of peroxynitrite is mediated by its capability to nitrate several molecules and to generate 3-NT, which has been proposed to be a relevant factor in the pathology of cardiovascular disease [15, 44]. The high levels of 3-NT in MUN rats can lead to vascular damage and participate in structural and functional alterations. In fact, we evidenced a marked reduction of vasodilator responses, suggesting endothelial dysfunction. MUN males and females showed reduced maximal responses to ACh. However, maximal relaxation was lower in MUN males, as shown by the significant interaction between group and sex, similar to what has been previously described in other rat models of fetal undernutrition [17–39, 45]. We used the iliac artery, a vessel in which, like the aorta, vascular relaxation is NO-dependent [35]. This was demonstrated in the present study by the abolition of acetylcholine responses by l-NAME. Only MUN males showed lower l-NAME responses compared to controls, which indicate that basal NO availability was reduced. The better responses in females may be related to the presence of sex hormones. Estrogens contribute to protection of females exposed to fetal insults against development of hypertension, interacting with the renin angiotensin system (RAS), as antioxidants and as eNOS activators increasing vasodilation [11]. MUN females showed a better antioxidant status and lower level of nitrosative damage in the present study. However, we demonstrated a certain degree of endothelial dysfunction and a tendency towards higher blood pressure levels in MUN females. This fact, together with our previous study showing hypertension development in MUN females in ageing [20], supports the protective effects of estrogens in this rat model of FGR. Besides, testosterone may also play a role; in models of fetal programing induced by placenta insufficiency, higher testosterone levels participate in hypertension programming. However, in animals exposed to undernutrition, no clear differences in circulating testosterone have been observed [1]. In a previous study, we showed a tendency towards higher levels of testosterone in MUN males at the age of 21 days [34]. It has been proposed that in fetal programming the effects of testosterone on hypertension development may be mediated by Ang II [1]. Since we have demonstrated that MUN male rats exhibit a disequilibrium in arterial RAS [41], it is possible that elevation of testosterone in early life may contribute through this pathway.

We found a marked increase in plasma NOx in MUN rats. Since local NO availability is likely reduced due to inactivation by superoxide anion, we suggest that the source could be iNOS. Several reports in models of fetal programming do not show that iNOS is elevated in the vascular wall [17, 18, 46]. However, other cell types, including immune cells, express iNOS when exposed to pro-inflammatory cytokines [5]. Therefore, the role of inflammation in this model of fetal programming deserves further investigation.

In summary, our data suggest that NO alterations in MUN male rats due to destruction by ROS and subsequent nitrosative damage, may contribute to vascular dysfunction and hypertension development. We cannot discard that other alterations in systems related to cardiovascular control, such as disbalanced RAS [37] or sympathetic hyperactivity [25], may also be implicated, since we have evidence of these disturbances in male MUN offspring [41, 42]. Females show a better adaptation to fetal undernutrition, particularly related to oxidative and nitrosative damage. However, the observed endothelial dysfunction may contribute to high blood pressure in ageing.

### Limitations of the study

We did not quantify BH4, an important co-factor of eNOS; the concentration of which is critical in the “redox switch” between NO and superoxide production by the enzyme [40]. It is possible that low concentrations of BH4 could favor higher superoxide levels contributing to oxidative and nitrosative damage. Secondly, we did not assess local NO-production, which can be obtained by fluorescent indicators, due to technical difficulties related to elastin interference. We used aorta and iliac arteries due to the key role of NO in vasodilatation. However, it would be interesting to evaluate vascular function in resistance vessels, which play a major role in blood pressure regulation. Finally, we only quantified Nrf2, and it would have been desirable to evaluate downstream antioxidant genes or its regulators, such as Keap-1 or Bach-1.

## Conclusion

In summary, our data suggest that interaction between excess vascular superoxide anion and NO, and associated nitrosative damage, may contribute to hypertension development in MUN males.

## Data Availability

Raw data can be provided to researchers interested on request to the corresponding author. Western blot data are provided as supplementary material.
